# Risk factors for acute respiratory distress syndrome during neutropenia recovery in patients with hematologic malignancies

**DOI:** 10.1186/cc8149

**Published:** 2009-11-03

**Authors:** Chin Kook Rhee, Ji Young Kang, Yong Hyun Kim, Jin Woo Kim, Hyung Kyu Yoon, Seok Chan Kim, Soon Suk Kwon, Young Kyoon Kim, Kwan Hyung Kim, Hwa Sik Moon, Sung Hak Park, Hee Je Kim, Seok Lee, Jeong Sup Song

**Affiliations:** 1 Division of Pulmonary and Critical Care Medicine, Department of Internal Medicine, College of Medicine, Catholic University of Korea, 505 Banpo-Dong, Seocho-Gu, Seoul 137-701, Korea; 2 Catholic Hematopoietic Stem Cell Transplantation Center, Department of Internal Medicine, College of Medicine, Catholic University of Korea, 505 Banpo-Dong, Seocho-Gu, Seoul 137-701, Korea

## Abstract

**Introduction:**

Neutropenia recovery may be associated with deterioration in oxygenation and exacerbation of pre-existing pulmonary disease. However, risk factors for acute respiratory distress syndrome (ARDS) during neutropenia recovery in patients with hematologic malignancies have not been studied.

**Methods:**

We studied critically ill patients with hematologic malignancies with the dual objectives of describing patients with ARDS during neutropenia recovery and identifying risk factors for ARDS during neutropenia recovery. A cohort of consecutive neutropenic patients with hematologic malignancies who were admitted to the intensive care unit (ICU) was studied. During a 6-year period, 71 patients recovered from neutropenia, of whom 38 (53.5%) developed ARDS during recovery.

**Results:**

Compared with non-ARDS patients, patients who experienced ARDS during neutropenia recovery were more likely to have pneumonia, be admitted to the ICU for respiratory failure, and receive mechanical ventilator therapy. The in-ICU mortality was significantly different between the two groups (86.8% versus 51.5%, respectively, for patients who developed ARDS during neutropenia recovery versus those who did not during neutropenia recovery). In multivariate analysis, only occurrence of pneumonia during the neutropenic episode was associated with a marked increase in the risk of ARDS (odds ratio, 4.76).

**Conclusions:**

Patients with hematologic malignancies complicated by pneumonia during neutropenia are at increased risk for ARDS during neutropenia recovery.

## Introduction

Over the past two decades, the survival of patients with a hematologic malignancy has substantially improved as a result of new and intensive chemotherapeutic regimens, which may be followed by hematopoietic stem cell transplantation (HSCT) [1]. Unfortunately, the use of aggressive chemotherapeutic regimens frequently results in life-threatening complications, requiring transfer to the intensive care unit (ICU) for monitoring or advanced support [2]. Respiratory failure is the most common reason for ICU admission in critically ill patients with hematologic malignancies [3].

Intensive chemotherapeutic treatment results in an increase in the number of patients with neutropenia. In cancer patients, neutropenia recovery may be associated with a deterioration in oxygenation and exacerbation of pre-existing pulmonary disease [4,5]. However, risk factors for acute respiratory distress syndrome (ARDS) during neutropenia recovery in a cohort of patients with hematologic malignancies have not been studied.

We studied a cohort of critically ill patients with hematologic malignancies with the dual objectives of describing patients with ARDS during neutropenia recovery and identifying risk factors for ARDS during neutropenia recovery.

## Materials and methods

We studied a cohort of consecutive neutropenic patients with hematologic malignancies who were admitted to the hematology ICU of St. Mary's Hospital (Seoul, Korea). In this hospital, more than 250 HSCTs are performed annually. The hematology ICU is equipped for neutropenic precautions and has laminar airflow with high-efficiency particulate air filtration. The study period was from March 2002 to August 2008. Patients were identified by medical record review. The institutional review board of St. Mary's Hospital approved the study. The requirement for informed consent from the patients studied was waived by the ethical review board.

Epidemiologic and clinical data were taken from the medical chart of each patient at ICU admission. Data included: gender; age; characteristics of the disease, including the type of malignancy, time since diagnosis, prior treatments, and current status; and whether ICU admission was for acute respiratory failure, shock, coma, or acute renal failure. We also noted therapeutic interventions during the stay in the ICU, including mechanical ventilation (MV), vasopressor treatment, dialysis, and administration of granulocyte colony-stimulating factor (G-CSF). MV was started with volume-assisted ventilation (tidal volume: 6 mL/kg of predicted body weight), then adjusted according to blood gas analysis by a critical care specialist.

In neutropenic patients and in patients undergoing neutropenia recovery, clinically documented pneumonia was defined as new pulmonary infiltrates with clinical manifestations that suggested pneumonia such as fever, tachypnea and/or blood gas deterioration [5]. X-ray and computed tomography scan images were confirmed by the radiologist to differentiate pneumonia from pulmonary edema. Microbiologically documented pneumonia was defined as an endotracheal aspirate culture showing more than 10^3 ^colony forming units/mL [5,6]. In blood culture, microbiologically documented pneumonia was accepted only when there was no other cause of sepsis than pneumonia. Complete remission was defined as: less than 5% of blast cells in marrow aspirates in leukemia patients; disappearance of peripheral and deep lymphadenopathy, and other malignant foci in lymphoma patients; and disappearance of monoclonal immunoglobulin in blood and urine and less than 5% plasma cells in bone marrow aspirates in myeloma patients. Neutropenia was defined as a leukocyte count of less than 1000 cells/mm^3^. Neutropenia recovery was defined as the seven-day period centered on the day the neutrophil count rose above 1000 cells/mm^3^[5]. ARDS was defined by the presence of three criteria: 1) partial pressure of oxygen in arterial blood (PaO_2_)/fractional concentration of inspired oxygen (FiO_2_) ratio of 200 mmHg or less; 2) bilateral alveolar or interstitial infiltrates; 3) pulmonary capillary wedge pressure (PCWP) of 18 mmHg or less, or no clinical evidence of increased left atrial pressure. For patients with more than one hospital admission during the study period, only the first admission was included in the analysis to ensure independence of the observations.

### Statistical analysis

All results are reported as means ± standard error of the mean (SEM) or frequencies (%). Patient characteristics were compared using the chi-squared test or Fisher's exact test, as appropriate, for categorical variables, and independent samples t-tests for continuous variables. Multivariate analysis was performed to investigate associations between patient characteristics and the occurrence of ARDS during neutropenia recovery. Odds ratios and their 95% confidence intervals were computed. Goodness of fit was computed to assess the relevance of the logistic regression model. All tests were two-sided, and *P *values of less than 0.05 were considered statistically significant. All statistical analyses were performed using SPSS software (Chicago, IL, USA).

## Results

**Figure 1:**
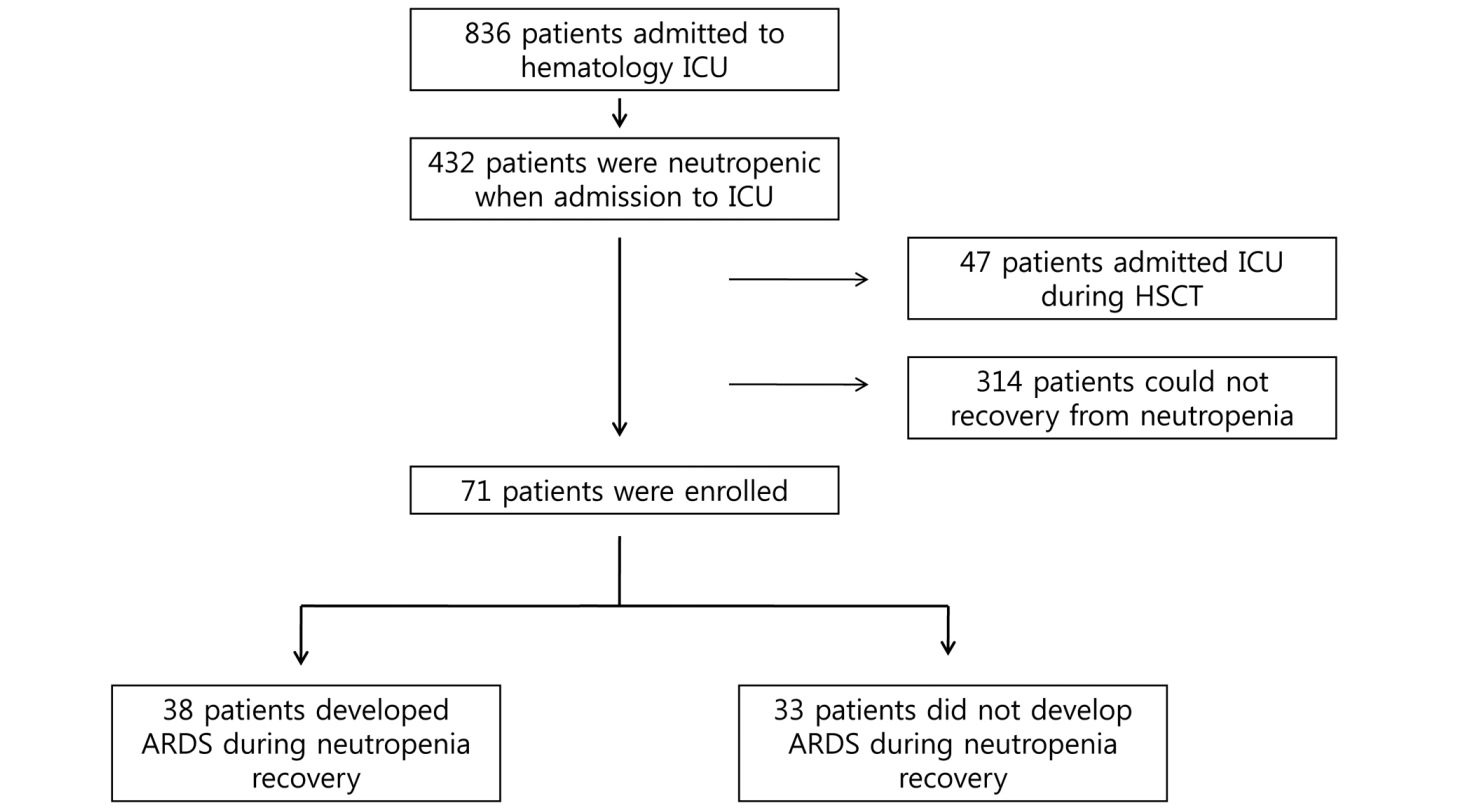
Study subjects. ARDS = acute respiratory distress syndrome; ICU = intensive care unit, HSCT = hematopoietic stem cell transplantation.

Among the 836 patients with hematologic malignancies admitted to our hematology ICU from March 2002 to August 2008, 432 (51.7%) were neutropenic. Of these 432 patients, 47 patients were admitted during HSCT, and 314 patients did not recover from the neutropenia. A total of 71 patients recovered from neutropenia during their ICU stay and were included in the study. Of these 71 patients, 38 (53.5%) developed ARDS during neutropenia recovery (Figure 1).

### Patient characteristics

Of the patients, 33 (46.5%) were men and 38 were women, with a median age of 45.71 years. The diagnosis was acute myeloid leukemia (AML) in 35 (49.3%) patients, acute lymphoblastic leukemia (ALL) in 22 (31%) patients, and lymphoma in 8 (11.3%) patients. The median duration of neutropenia was 22.54 days. During neutropenia, pneumonia developed in 45 (63.4%) patients. Among them, 17 (37%) patients had microbiological documentation and 3 (6.7%) patients had aspiration pneumonia. MV was needed in 53 (74.6%) patients. A total of 50 (70.4%) patients died during the ICU stay (Table 1). All neutropenic patients received G-CSF.

**Table 1:** Characteristics of the 71 patients with hematologic malignancies who recovered from neutropenia during intensive care unit stay

Characteristic	No (%) or mean ± SEM
Age, year	45.71 ± 1.50
Male	33 (46.5%)
AML	35 (49.3%)
ALL	22 (31.0%)
Lymphoma	8 (11.3%)
Multiple myeloma	4 (5.6%)
Relapsed malignancy	17 (23.9%)
History of HSCT	13 (18.3%)
Neutropenia duration, days	22.54 ± 1.65
ARDS during neutropenia recovery	38 (53.5%)
Pneumonia during neutropenia	45 (63.4%)
Mechanical ventilation	53 (74.6%)
Renal replacement therapy	21 (29.6%)
In-ICU mortality	50 (70.4%)

AML = acute myeloid leukemia; ALL = acute lymphoblastic leukemia; ARDS = acute respiratory distress syndrome; HSCT = hematopoietic stem cell transplantation; ICU = intensive care unit; SEM = standard error of the mean.

### Comparison of patients with and without ARDS during neutropenia recovery

**Figure 2:**
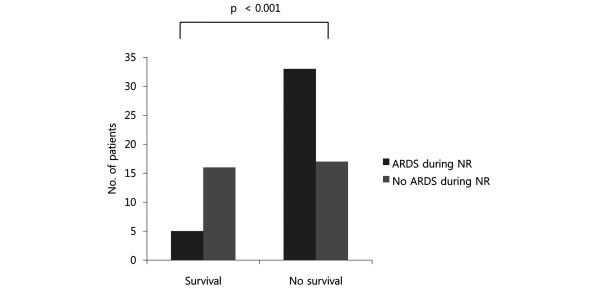
Number of patients who survived or died during intensive care unit stay. ARDS = acute respiratory distress syndrome; NR = neutropenia recovery.

**Figure 3:**
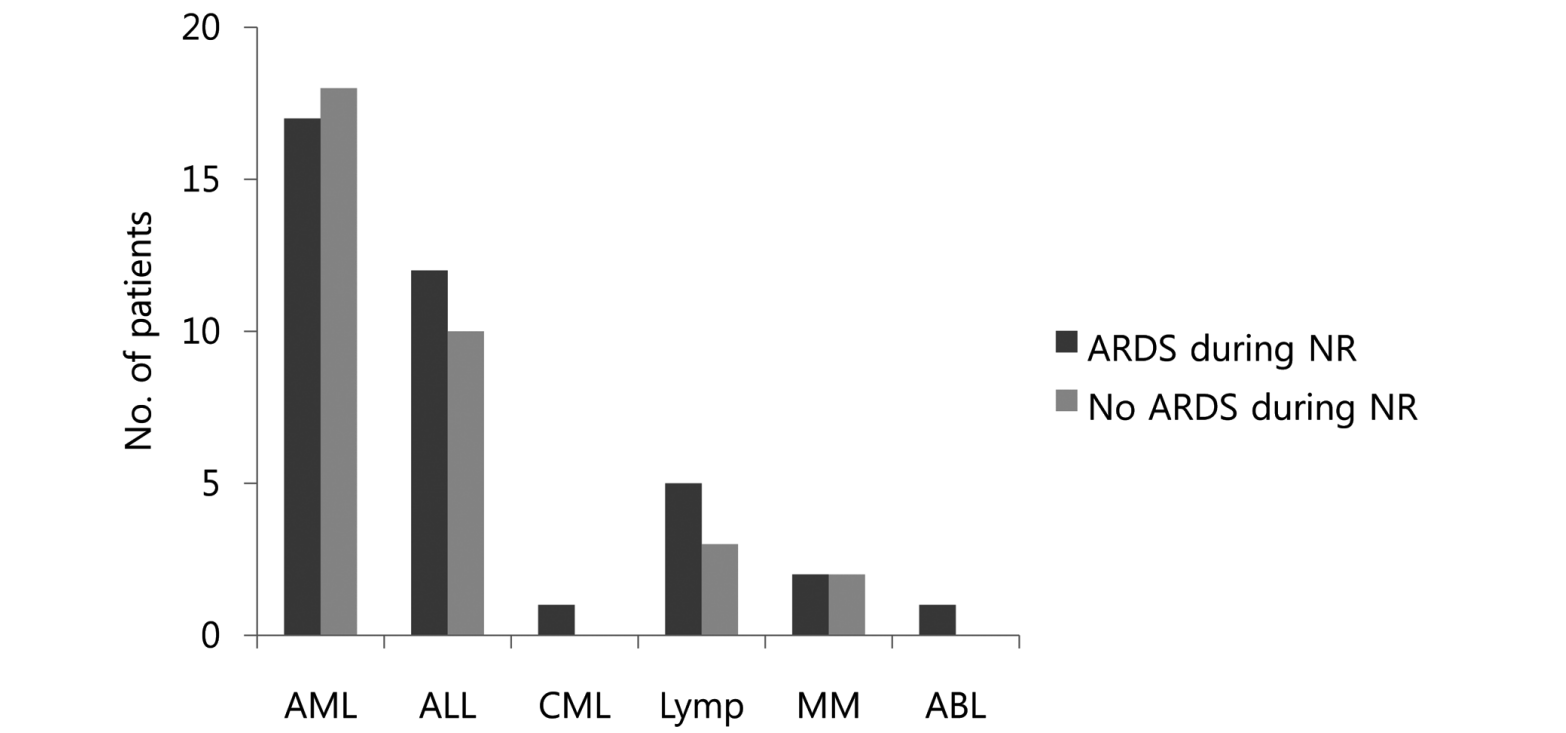
Number of patients according to underlying disease. ABL = acute biphenotypic leukemia; AML = acute myeloid leukemia; ALL = acute lymphoblastic leukemia; ARDS = acute respiratory distress syndrome; CML = chronic myeloid leukemia; Lymp = lymphoma; MM = multiple myeloma; NR = neutropenia recovery.

Table 2 shows the results of the univariate analyses. The in-ICU mortality was significantly different between the two groups (86.8% vs. 51.5%, respectively, for ARDS during neutropenia recovery vs. no ARDS during neutropenia recovery; Figure 2). There were no significant differences between the two groups in underlying diseases (Figure 3), total duration of chemotherapy, or duration of neutropenia. Time between neutropenia recovery and onset of ARDS in the ARDS during neutropenia recovery group was -0.95 ± 0.58 days (mean ± SEM). All patients had respiratory signs one or two days before neutropenia recovery in the ARDS during neutropenia recovery group, while 21 (63.6%) patients had no signs in the ARDS during neutropenia recovery group. Among the patients who developed ARDS during neutropenia recovery, 32 (84.2%) had pneumonia during neutropenia. Among them, 13 (40.6%) had microbiological documentation. The organisms involved were *Pseudomonas aeruginosa *(n = 2), *Escherichia coli *(n = 3), *Staphylococcus aureus *(n = 2), *Klebsiella pneumoniae *(n = 2), *Staphylococcus epidermidis *(n = 1), *Enterococcus faecalis *(n = 2), and *Enterococcus faceum *(n = 1). Patients who experienced ARDS during neutropenia recovery were more likely to have pneumonia, be admitted to the ICU for respiratory failure, and receive MV therapy. When the three variables that were significant in the univariate analysis were introduced into a logistic regression model, only one was independently associated with ARDS (Table 3). Occurrence of pneumonia during the neutropenic episode was associated with a marked increase in the risk of ARDS (odds ratio, 4.76).

**Table 2:** Comparison of patients with and without ARDS during neutropenia recovery

	ARDS during NR	No ARDS during NR	Odd ratio	95% CI	*P *value
Age	45.99 ± 1.79	45.38 ± 2.51			0.84
Male	20 (52.6%)	13 (39.4%)	1.71	0.66-4.40	0.27
AML	17 (44.7%)	18 (54.5%)	0.68	0.26-1.72	0.41
ALL	12 (31.6%)	10 (30.3%)	1.06	0.39-2.91	0.91
Relapsed maligancy	10 (26.3%)	7 (21.2%)	1.33	0.44-3.99	0.62
Time from diagnosis, days	244 ± 98.7	140 ± 56.8			0.38
Anthracycline treatment	20 (52.6%)	18 (54.5%)	0.93	0.36-2.36	0.87
Cyclophosphamide treatment	9 (23.7%)	5 (15.2%)	1.74	0.52-5.83	0.37
Methotrexate treatment	6 (15.8%)	3 (9.1%)	1.88	0.43-8.12	0.49
Total chemotherapy times	2.79 ± 0.27	2.36 ± 0.25			0.26
Total chemotherapy times ≥3 times	20 (52.6%)	14 (42.4%)	1.51	0.59-3.86	0.39
Previous history of HSCT	8 (21.1%)	5 (15.2%)	1.88	0.43-8.18	0.52
Time between chemotherapy and onset of neutropenia	3.45 ± 0.69	2.18 ± 0.65			0.19
Time between chemotherapy and onset of neutropenia >10 days	3 (7.9%)	1 (3.0%)	2.74	0.27-27.73	0.62
Neutropenia duration, days	22.4 ± 2.3	22.7 ± 2.4			0.94
Neutropenia duration >10 days	34 (89.5%)	26 (78.8%)	2.29	0.61-8.66	0.22
Platelet transfusion during neutropenia	38 (100%)	32 (97%)			0.47
Plasma transfusion during neutropenia	33 (86.8%)	26 (78.8%)	1.78	0.51-6.25	0.37
Pneumonia during neutropenia	32 (84.2%)	13 (39.4%)	8.21	2.68-25.07	< 0.001
RRT during neutropenia	13 (34.2%)	8 (24.2%)	1.63	0.57-4.60	0.36
ICU admission for respiratory failure	25 (65.8%)	10 (30.3%)	4.42	1.63-12.03	0.003
Mechanical ventilator therapy	34 (89.5%)	19 (57.6%)	6.26	1.80-21.75	0.002
In-ICU mortality	33 (86.8%)	17 (51.5%)			< 0.001

AML = acute myeloid leukemia; ALL = acute lymphoblastic leukemia; ARDS = acute respiratory distress syndrome; CI = confidence interval; HSCT = hematopoietic stem cell transplantation; ICU = intensive care unit; NR = neutropenia recovery; RRT = renal replacement therapy.

**Table 3:** Multivariate analysis of patient characteristics

	Odds ratio	95% CI	*P *value
Mechanical ventilator	3.68	0.91-14.82	0.067
Pneumonia during neutropenia	4.76	1.41-16.00	0.012
ICU admission for respiratory failure	2.44	0.79-7.57	0.121

ICU = intensive care unit. Goodness of fit (Hosmer-Lemeshow) chi-squared *P *value = 0.904.

## Discussion

Neutropenia recovery increases the risk of deterioration of oxygenation and abnormal lung microvascular permeability [4]. A few case reports have been published about this relationship [7,8]. However, the small number of reported cases leaves room for doubt about the association of neutropenia with these conditions. Azoulay and colleagues [5] showed that in 62 critically ill cancer patients recovering from neutropenia, recovery was associated with development of ARDS. However, the patients were relatively heterogeneous and included solid cancer patients. In our study, we enrolled only hematologic malignancy patients, and we excluded patients who were admitted to the ICU during HCST because these patients show relatively different clinical manifestations and usually have a poor prognosis [9-11]. Despite these limitations, the number of patients enrolled in our study (n = 71) was higher than that of the previous study (n = 62) [5]. Thus, in a single homogenous cohort, we showed that recovery from neutropenia might be associated with the development of ARDS.

Although there is some debate about the association, five points support a link between ARDS and neutropenia recovery. i) Among enrolled patients, 38 of 71 (53.5%) patients experienced ARDS during neutropenia recovery, a proportion higher than that reported during sepsis and pancreatitis, two widely recognized risk factors for ARDS [12-14]. ii) Among 43 patients with ARDS, 38 (88.4%) patients developed ARDS during neutropenia recovery period, while only 5 (11.6%) patients developed before or after neutropenia recovery (*P *< 0.001). iii) ARDS during neutropenia recovery has been reported by other groups [4-8,15,16]. iv) Exacerbation of prior acute lung disease during neutropenia recovery has been demonstrated by animal models [17]. v) The condition of biological plausibility is met, because cancer patients are at risk for lung injury caused by pulmonary toxicity from chemotherapeutic agents [18-21] and G-CSF [22] and/or to pneumonia associated with immunodeficiency [23].

Compared with solid cancer patients, patients with hematologic malignancies have a longer duration of neutropenia because the dose of chemotherapeutic agents used in the treatment of hematologic tumors is much higher than that for solid cancers, and the function of bone marrow is also decreased. In addition, the incidence of neutropenia is much higher due to malignant infiltration of the bone marrow. Moreover, neutrophil function is defective because many neutrophils are malignant cells. Therefore, the nature of neutropenia recovery in patients with hematologic malignancies is very different from that in patients with solid cancers.

We excluded from the study patients who developed neutropenia during HSCT. In HSCT, more intense chemotherapy is applied than in induction or consolidation chemotherapy, and total body irradiation is also given, so the duration of neutropenia is much longer and the defect in immunity is more profound. Thus, the clinical aspects are very different from those of post-chemotherapy neutropenic patients with hematologic malignancies.

In a single-cohort study, we showed that the occurrence of pneumonia was a strong risk factor for developing ARDS. This result is similar to that of the previous study [5], and the odds ratio is also similar (4.15 vs. 4.76). However, contrary to the results of that study, a period of more than 10 days between chemotherapy and the onset of neutropenia, and duration of neutropenia of more than 10 days were not risk factors in our study. This difference may arise from the different nature of the enrolled patients. In the study by Azoulay and colleagues [5], the numbers of patients with solid cancers and lymphoma were high, but those numbers were low in our study. Instead, the numbers of AML and ALL patients were high in our study. In AML and ALL patients, the period between chemotherapy and the onset of neutropenia is usually short compared with that in solid cancer or lymphoma patients. Some of the AML and ALL patients were already in a neutropenic state at the start of chemotherapy. AML and ALL patients also had a longer duration of neutropenia. In the study by Azoulay and colleagues [5], 12 (19%) of 62 patients had a period of more than 10 days between chemotherapy and the onset of neutropenia. In our study, only 4 (6%) of 71 patients had a period of more than 10 days. In the study by Azoulay and colleagues [5], the number of patients with a duration of neutropenia of more than 10 days was 28 (45%). In our study, the number was 60 (85%).

Relapsed malignancy is associated with poor prognosis in hematologic malignancy patients. In a study of mortality among patients admitted to the ICU with hematologic malignancies, mortality among 22 patients with relapsed malignancies (21 deaths) was significantly higher than among 35 patients at first presentation (26 deaths) [24]. Crawford and colleagues analyzed the risk factors for and the outcome of MV support after bone marrow transplantation in 1089 consecutive bone marrow recipients. A multivariate regression model revealed that hematologic malignancy in relapse was associated with ventilator support [25]. However, in our study, there was no significant difference in relapsed malignancy between patients with and without ARDS during neutropenia recovery.

Although the pathophysiology of ARDS is controversial, there is abundant evidence that neutrophil recruitment to and activation in the lung may play a key role [26]. Lungs damaged by chemotherapy and infection may be particularly sensitive to the influx of neutrophils that probably accompany neutropenia recovery [5,27]. Terashima and colleagues [28] showed that younger neutrophils released from the bone marrow are preferentially sequestered in pulmonary microvessels and may contribute to the alveolar wall damage seen in ARDS. Moreover, the same group reported that pneumonia shortened the transit time of neutrophils in the marrow, which may result in the release of immature neutrophils with higher levels of lysosomal enzymes into the circulation [29].

During neutropenia recovery, alveolar macrophages remained the predominant cells in bronchoalveolar lavage (BAL) fluid [5]. So, alveolar macrophages may be responsible for lung injury in the context of alveolar neutropenia. Mokart and colleagues [30] showed deactivation of alveolar macrophages in septic neutropenic ARDS. They also showed monocyte deactivation in neutropenic ARDS patients [31]. Although exact pathogenesis is unknown, deactivation of macrophages during neutropenia may be related to development of ARDS.

G-CSF is widely used in hematologic malignancy patients to reduce the duration of chemotherapy-induced neutropenia. In these circumstances, G-CSF allows for closer spacing of chemotherapy courses, thereby substantially improving prognosis [32,33]. Although G-CSF is generally safe and well tolerated, there have been several reports of acute respiratory failure during G-CSF-induced neutropenia recovery [15,34]. G-CSF upregulates the production of cytokines that increase alveolar permeability and neutrophil influx, such as TNF-α, IL-1β, and IL-8 [35,36]. *In vitro *studies have also found enhanced secretion of proinflammatory cytokines by alveolar macrophages isolated during neutropenia recovery from rats that received G-CSF, compared with rats that did not, providing a possible explanation for the exacerbation of lung injury during G-CSF-induced recovery from neutropenia [17]. The authors concluded that neutropenia recovery could worsen acute lung injury, and this effect could be exacerbated by G-CSF [17]. Moreover, in a clinical study, Karlin and colleagues showed that G-CSF-induced neutropenia recovery was associated with a risk of deterioration in respiratory status [6]. Because all the patients in our study received G-CSF, this association would definitely contribute to the development of ARDS during neutropenia recovery.

Azoulay and colleagues reported 84 cases with probable G-CSF-related pulmonary toxicity among 1801 patients receiving G-CSF treatment [15]. In that review, ARDS was prone to develop in patients who had a history of more than three courses of chemotherapy. In our study, however, there was no significant difference in the total number of courses of chemotherapy between patients with and without ARDS during neutropenia recovery.

Our study has some limitations. First, this was not a prospective study. All data were obtained from retrospective review of medical records. However, in a single cohort whose treatment was based on the same protocol [37-39], we carefully inspected all the patients who were admitted to the hematologic ICU and were enrolled in the study. The aim of this study was to identify risk factors for developing ARDS during neutropenia, so the setting of our study may not have differed greatly from that of a prospective observational study.

Second, the number of patients enrolled was small. However, the number of patients with hematologic malignancies who are admitted to the ICU is low, and the incidence of ARDS during neutropenia recovery is even lower. St. Mary's Hospital is one of the largest HSCT centers in Asia. To the best of our knowledge, the number of patients with this condition enrolled in our study was the largest of any such study to date, so our data may be representative of this disease group. Third, our study showed relatively high mortality compared with a previous study [5] (86.8% vs. 61.9% in patients with ARDS during neutropenia recovery, and 39% vs. 51.5% in patients without ARDS during neutropenia recovery, respectively). This may arise from the different diseases of the enrolled patients. All of the patients in our study had hematologic malignancies, especially AML and ALL. Moreover, many patients had factors associated with poor prognosis, such as chromosomal aberrations or relapsed malignancies, and these factors definitely contributed to the high mortality rate.

Fourth, the diagnostic criteria of pneumonia may not have been explicit in our study. However, all the patients who were classified as having pneumonia had clinical manifestations that suggested pneumonia. Fever, tachypnea, dyspnea, oxygen saturation deterioration, and elevated C-reactive protein level were observed in all patients. X-ray findings and CT scan images were confirmed by the radiologist to differentiate pneumonia from other diseases. Bronchoscopy was not performed because of the poor clinical condition of the patients, risk of hypoxemia, and potential for opportunistic infection. However, cultures of endotracheal aspirates and blood were performed in almost all patients and resulted in 37% of microbiological documentation. In spite of the lack of BAL, this result was comparable with a previous study with BAL (17/45 vs. 13/29) [5]. These data suggest that most patients who were classified as pneumonia in this study actually were pneumonia patients.

## Conclusions

In the present study, we found that the main risk factor for ARDS during neutropenia recovery in hematologic malignancy patients was the occurrence of pneumonia. In patients with hematologic malignancies who have pneumonia during neutropenia, close monitoring of respiratory status during neutropenia recovery is very important. When pulmonary infiltrate is noted and respiratory symptoms exist before neutropenia recovery, early respiratory care should be offered. Further study is needed of patients with hematologic malignancies who have ARDS during neutropenia.

## Key messages

• Patients with hematologic malignancies who experienced ARDS during neutropenia recovery were more likely to have pneumonia, be admitted to the ICU for respiratory failure, and receive mechanical ventilator therapy.

• Patients with hematologic malignancies who experienced ARDS during neutropenia recovery showed higher mortality than those who did not during neutropenia recovery.

• Patients with hematologic malignancies complicated by pneumonia during neutropenia are at increased risk for ARDS during neutropenia recovery.

## Abbreviations

ALL: acute lymphoblastic leukemia; AML: acute myeloid leukemia; ARDS: acute respiratory distress syndrome; BAL: bronchoalveolar lavage; FiO_2_: fractional concentration of inspired oxygen; G-CSF: granulocyte colony-stimulating factor; HSCT: hematopoietic stem cell transplantation; ICU: intensive care unit; IL: interleukin; MV: mechanical ventilation; PaO_2_: partial pressure of oxygen in arterial blood; PCWP: pulmonary capillary wedge pressure; SEM: standard error of the mean; TNF: tumor necrosis factor.

## Competing interests

The authors declare that they have no competing interests.

## Authors' contributions

CKR, JYK, YHK, JWK, HKY and SCK collected and analyzed the data. CKR, SSK, YKK, KHK, HSM, SHP, and JSS reviewed the study. HJK, and SL coordinated the study.
